# Transforming One Health in India: National Multisectoral Mixed Method Study on Prioritization of Zoonotic Diseases

**DOI:** 10.2196/77850

**Published:** 2025-12-22

**Authors:** Simmi Tiwari, Indranil Roy, Monal Daptardar, Ruchi Singh, Anshuman Mishra, Richa Kedia, Ananta Bhat, Harmesh Manocha, Mayank Dwivedi, Amlesh Dwivedi, Gaurish Shukla, Ajit Shewale, Tushar Nale, Dipti Mishra, Ravi Prakash Sharma, Daniel Garcia, Runa Hatti Gokhale, Meghna Desai, Sujeet Singh

**Affiliations:** 1 National Center for Disease Control New Delhi India; 2 Centers for Disease Control and Prevention (CDC), India New Delhi India; 3 Directorate of Medical and Health Services, Jaipur, Rajasthan, India Jaipur India; 4 American Society for Microbiology Washington DC, WA United States; 5 Society for Health Allied Research and Education New Delhi India; 6 Government Institute of Medical Sciences (GIMS), Greater Noida, Uttar Pradesh, India Greater Noida India

**Keywords:** One Health, One Health Zoonotic Disease Prioritization, OHZDP, zoonosis, multisectoral collaboration, disease prioritization

## Abstract

**Background:**

To tackle the risk of emerging and re-emerging diseases, it is critical for countries with limited resources to prioritize endemic and emerging zoonotic diseases of greatest national concern. One Health is an integrated, unifying approach that aims to sustainably balance and optimize the health of people, animals, and ecosystems.

**Objective:**

In India, as a first step toward a multidisciplinary, multisectoral, One Health approach to preventing and detecting zoonotic disease outbreaks, a national-level multistakeholder zoonotic disease prioritization workshop was organized to identify a list of zoonotic diseases of greatest national concern for India.

**Methods:**

We followed the Good Reporting of a Mixed Methods Study guidelines to finalize a list of priority zoonotic diseases through a participatory action research approach involving 50 experts in zoonotic diseases. We used a prioritization process based on the US Centers for Disease Control and Prevention’s semiquantitative One Health Zoonotic Disease Prioritization process, with modifications per country need.

**Results:**

We ranked 40 zoonotic diseases based on 5 criteria: severity of illness in humans, the economic burden of the diseases, pandemic potential, capacity for prevention and control, and potential for introduction or increased transmission in India. The final list of zoonotic diseases ranked in the order of national significance included the following top 10 priority zoonotic diseases: zoonotic influenza (zoonotic influenza A viruses), anthrax, Japanese encephalitis, leptospirosis, brucellosis, dengue fever, rabies, scrub typhus, plague, and Crimean-Congo hemorrhagic fever. We conducted a sensitivity analysis to assess the impact of each criterion on the prioritized list; this analysis showed minimal changes in ranking for the top 10 diseases.

**Conclusions:**

For the successful adoption of One Health practices in India, multisectoral collaboration is critical at all levels—national, state, and provincial. This collaborative prioritization process conducted at the national level has the potential to fast track India’s one health efforts and enhance zoonotic disease prevention and detection efforts at the state and local levels across India.

**International Registered Report Identifier (IRRID):**

RR2-10.1101/2024.02.26.24303393

## Introduction

India is one of the 12 mega biodiverse countries in the world, with 11% of the world’s flora in approximately 2.4% of its land mass [1]. Intensification of agriculture, rapid urbanization, and population growth have altered ecosystems such that the natural balance is inclined toward increased animal-human association [2], which brings increased risk to wildlife and humans from emerging and re-emerging infectious diseases [3,4]. As such, India has been identified as a hot spot for the transmission of both known and novel infectious agents between animals and people [5].

One Health is an integrated, unifying approach that aims to sustainably balance and optimize the health of people, animals, and ecosystems. It recognizes that the health of humans, domestic and wild animals, plants, and the wider environment (including ecosystems) is closely linked and interdependent [6]. Zoonoses (infections that are transmitted between animals and humans) are an important cause of human illness worldwide. It has been observed that as much as 40% of morbidity and mortality caused by diseases in low-income countries can be attributed to infectious diseases, up to one-fifth of which can be due to zoonotic diseases [7]. India has a diverse geography with large forest covers and has experienced repeated outbreaks of a wide range of zoonotic diseases, including Crimean-Congo hemorrhagic fever (CCHF), Kyasanur forest disease, and Nipah virus, which are both lethal and uncommon in other parts of the world. The COVID-19 pandemic, which was believed to have an animal origin, caused an unprecedented level of morbidity and mortality worldwide and fulfilled the prediction of global health experts that another pandemic with the speed and severity of the 1918 influenza epidemic was a matter “not of if, but of when” [8]*.* Addressing such a challenge will need a One Health approach to help understand and mitigate the risks that exist at the interface among humans, animals, and their environments [9].

A robust understanding of the pathogen ecology of natural host and human-host interactions is required to inform surveillance and public health interventions for preventing or mitigating future zoonotic spillover events [10]. Prioritization is required to ensure the strengthening of public health systems and efficient use of existing resources through a collaborative, multisectoral, transdisciplinary One Health approach [11]. Zoonotic disease prioritization enables countries to plan and calibrate their activities through a coordinated effort across the human, animal, wildlife, and environmental health sectors. The National Centre for Disease Control (NCDC) in India has already established a Centre for One Health, which is creating a countrywide network of sentinel surveillance sites for zoonotic diseases and working across sectors to move ahead with One Health action plans. Zoonotic disease prioritization involves the use of various tools incorporating qualitative, semiquantitative, and quantitative methods [12-19]. The US Centers for Disease Control and Prevention’s (CDC) One Health Zoonotic Disease Prioritization (OHZDP) process is a tool used to identify priority zoonotic diseases and develop next steps and action plans to tackle them [20].

In India, zoonotic infectious diseases such as cholera (*Vibrio cholerae* serogroup O139), sylvatic plague, Nipah virus disease, diphtheria, Chandipura virus disease, chikungunya, highly pathogenic avian influenza A (H5N1), novel influenza A (H1N1), CCHF, leptospirosis, anthrax, kala-azar, scrub typhus, acute encephalitis syndrome, and Kyasanur forest disease are reported through the Integrated Disease Surveillance Programme (IDSP). The IDSP is a national health program established in 2009 designed to strengthen and maintain a decentralized laboratory-based disease surveillance system for epidemic-prone diseases, monitor disease trends, and detect and respond to outbreaks [21]. Disease outbreaks reported in India include rabies, Chandipura virus disease (*Rhabdoviridae*; 1965, 2003, 2004, and 2007) [22,23], and chikungunya (1963 and 1973) [24].

A modified OHZDP process was used in India in 2019 for the prioritization of zoonotic diseases specific to the Indian city of Ahmedabad [25]; however, there has not been a similar prioritization at the national level. As a first step toward a national One Health approach to the prevention and detection of zoonotic diseases, a national-level multistakeholder, multisectoral zoonotic disease prioritization workshop was organized in Jaipur, in the state of Rajasthan, by the provincial health department of Rajasthan and the NCDC, government of India, with the support of the US CDC. The objectives of the workshop were to identify a list of zoonotic diseases of greatest national concern for India and develop cohesive, sustainable strategies for the prevention and control of these priority zoonoses.

## Methods

### Overview

**Figure 1:**
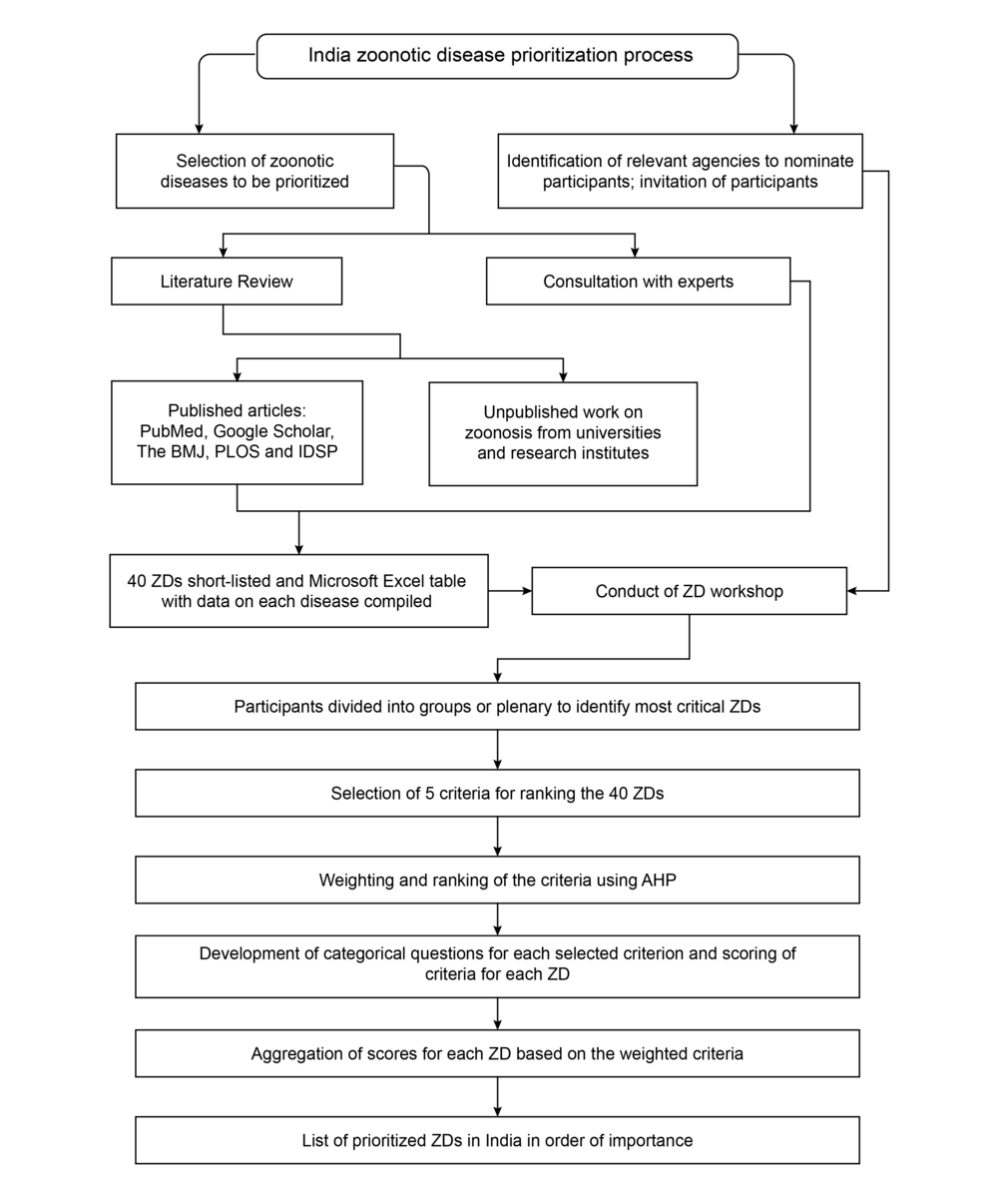
Flow of the workshop. AHP: analytic hierarchy process; IDSP: Integrated Disease Surveillance Programme; PLOS: Public Library of Science; ZD: zoonotic disease.

This study to finalize a list of priority zoonotic diseases for India through a participatory action research approach followed the Good Reporting of a Mixed Methods Study guidelines [26]. A mixed methods approach was necessary to incorporate both the qualitative and quantitative components adopted before, during, and after the 3-day national multistakeholder workshop (Figure 1). Discussions involved 50 experts in zoonotic diseases (Figure 1) from institutions including the Ministry of Fisheries, Animal Husbandry, and Dairying; the Ministry of Agriculture and Farmers’ Welfare; the Ministry of Environment, Forest, and Climate Change; the Ministry of Health and Family Welfare; state governments; private agencies; and national and international organizations (Textbox 1).

**List of participating institutions.**

- Calcutta School of Tropical Medicine, West Bengal
- Centers for Disease Control and Prevention India
- Central Zoo Authority, Delhi
- Department of Animal Husbandry and Dairying, Delhi
- Department of Animal Husbandry and Dairying, Rajasthan
- Department of Environment and Climate Change, Rajasthan
- Department of Microbiology, Government Medical College, Maharashtra
- Department of Microbiology, Mahatma Gandhi Institute of Medical Sciences, Maharashtra
- Department of Veterinary Biotechnology, Indian Veterinary Research Institute (IVRI), Uttar Pradesh
- Department of Veterinary Public Health, IVRI, Uttar Pradesh
- Department of Wildlife Science, Tamil Nadu University of Veterinary and Animal Sciences, Tamil Nadu
- Division of Animal Health, Indian Council of Agricultural Research (ICAR) research complex for North-Eastern Hill region, Meghalaya
- Government Institute of Medical Sciences, Uttar Pradesh
- ICAR-National Research Centre on Meat, Telangana
- Indian Association of Medical Microbiologists
- Integrated Disease Surveillance Programme, Gujarat
- Integrated Disease Surveillance Programme, Rajasthan
- Kasturba Medical College, Karnataka
- Maulana Azad Medical College, Delhi
- Mayo Hospital, Uttar Pradesh
- National Centre for Disease Control, New Delhi
- ICAR-National Institute of High Security Animal Diseases, Madhya Pradesh
- ICAR-National Institute of Veterinary Epidemiology and Disease Informatics, Karnataka
- ICAR-National Research Centre on Equines, Haryana
- Postgraduate Institute of Medical Education and Research, Chandigarh
- Public Health Foundation of India
- Regency Hospital, Uttar Pradesh
- School of Public Health and Zoonoses, Guru Angad Dev Veterinary and Animal Sciences University, Punjab
- Sawai Man Singh Medical College, Jaipur
- State Disease Diagnostic Laboratory, Department of Animal Husbandry, Rajasthan
- The American Society for Microbiology, United States
- Wildlife Institute of India, Dehradun
- World Health Organization, Rajasthan

The US CDC’s OHZDP process [20] was modified for country-specific requirements and used for this prioritization. The modified prioritization process was completed using the following 6-step sequence of methods.

### Step 1: Selection of Experts From the Field and Potential Priority Zoonotic Diseases

Experts were identified by first determining stakeholder organizations. A stakeholder was any organization, institution, network, or group involved in activities pertaining to zoonotic diseases at the national or international level. Initially, a formal communication was sent to state health departments, the Department of Animal Husbandry (Ministry of Fisheries, Animal Husbandry, and Dairying), the Department of Wildlife (Ministry of Environment, Forest, and Climate Change), state medical colleges, the Indian Council of Agricultural Research (Ministry of Agriculture and Farmers’ Welfare), the Indian Council of Medical Research (Ministry of Health and Family Welfare), veterinary universities, and international agencies in India to nominate or identify experts from these sectors at the provincial and federal level. Experts from these agencies were identified for participation in the workshop and were categorized as voters (n=33), advisors (n=10), and facilitators (n=7). In total, participation spanned seven major sectors. Doctors from clinical and diagnostic sectors constituted the largest group of experts (16, 32%), followed by veterinarians (12, 24%). Representatives from national-level public health institutions comprised 7 (14%) of participants, while state government officials and wildlife sector experts accounted for 5 (10%). International agencies represented 8 (16%) of the experts, and 2 (4%) were from the environment and food safety sector. The roles and responsibilities of each of these categories were as follows:

- Voting members—voting members were subject matter experts from the government public health, veterinary, wildlife, environment, and food sectors, as well as from international and private agencies. They were individuals with technical knowledge and programmatic experience in the field of zoonotic diseases. Their role during the workshop was to provide key inputs as per the selected criteria and support the prioritization of zoonotic diseases.
- Advisors—advisors were senior administrators and technical experts working in the public health, veterinary, wildlife, environment, and food sectors. They provided expertise during discussions with voting members to develop a multisectoral One Health zoonotic disease prevention, detection, and response plan for India.
- Facilitators—facilitators were experts with advanced computer skills and an understanding of the various methods used for prioritization, including the analytic hierarchy process (AHP). They steered the decision-making of voting members through objective, evidence-based data gathered from a literature review. AHP is a multicriteria decision-making method that is flexible and easy to use. It is used to identify or prioritize the net benefit of health interventions [27], in this case the prioritization of zoonotic diseases according to their overall impact on human life.

Three rounds of preworkshop online discussions were held to acquaint the voting members, advisors, and facilitators with the steps, process, and expected outcomes of the workshop. During these discussions, a list of 40 potential priority zoonoses was developed through a focused literature review and discussions (Textbox 2). The literature review included reports available under government health programs; peer-reviewed publications indexed on PubMed, Google Scholar, The BMJ, and Public Library of Science; and unpublished work on zoonoses from universities, research institutes and information created by government agencies and national programs such as the IDSP with publication dates from January 2000 to January 2020 (using the search terms “zoonotic disease” OR “zoonoses” OR “infectious disease” AND “India”).

**List of zoonotic diseases considered for prioritization.**

- Rabies
- Anthrax
- Brucellosis
- Plague
- Leptospirosis
- Scrub typhus
- Zoonotic influenza (zoonotic influenza A viruses)
- Kyasanur forest disease
- Crimean-Congo hemorrhagic fever
- Nipah virus infection
- Japanese encephalitis
- Toxoplasmosis
- Campylobacteriosis
- Lyme disease
- Echinococcosis
- Dengue fever
- Bartonellosis (cat-scratch disease)
- Psittacosis
- Tularemia
- Cryptococcosis
- Histoplasmosis
- Zoonotic tuberculosis
- Salmonellosis
- Melioidosis
- Glanders
- Cysticercosis
- Schistosomiasis
- West Nile fever
- Q fever
- Leishmaniasis
- Listeriosis
- Trypanosomiasis
- Middle East respiratory syndrome–related coronavirus
- Severe acute respiratory syndrome
- Ebola virus disease
- Yellow fever
- COVID-19
- Lassa fever
- Shiga toxin–producing
  Escherichia coli
- Blastomycosis

### Step 2: Development of Criteria for Ranking the Zoonotic Diseases

Before the workshop, the advisors and facilitators and most of the voting members identified in step 1 debated the criteria to be included, the supporting questions and responses, and the scores until consensus was achieved. Through this process, 5 criteria, along with categorical questions that would enable weighting and ranking for prioritization of zoonotic diseases, were decided upon. Prioritization documents produced by other countries were used as a reference. The discussion was led by the NCDC. The 5 criteria that were developed were the severity of the disease in humans, the economic burden of the disease, pandemic or epidemic potential, potential of transmission, and capacity for prevention and control in humans and the animal health sector. These criteria were presented at the workshop for discussion and ultimately used during the prioritization process (Table 1).

**Table 1:** Criteria, questions, responses, and scores finalized for ranking of zoonotic diseases.

Criterion	Criterion description	Questions	Response options and score
1	Severity of the disease in humans	Is the disease present in India? What is the CFR^a^ in humans?	Disease present, high CFR (≥5%; score of 3) Disease present, low CFR (<5%; score of 2) Disease not known to be present, high CFR (≥5%; score of 1) Disease not known to be present, low CFR (<5%; score of 0)
2	Capacity for prevention and control	Do measures exist for prevention and for control?	Both prevention and control capacity (score of 3) Prevention but no capacity for control (score of 2) No prevention but the capacity for control exists (score of 1) Neither prevention nor control capacity (score of 0)
3	Potential for introduction or increased transmission in India	(1) Does the disease have a feasible transmission pathway to India? (2) Has it been detected in India? (3) Has there been detection and spread in 5 or more new countries, regions, or states?	The response to all 3 questions is yes (score of 3) The response to 2 of the 3 questions is yes (score of 2) The response to 1 of the 3 questions is yes (score of 1) The response to none of the questions is yes (score of 0)
4	Economic burden of the disease	Does the disease cause economic impacts (variables include trade restrictions, decreased animal production, impact on outdoor recreation or tourism, intervention costs, or other secondary impacts [ecological impact])?	High (3 or more sectors face economic impacts; score of 3) Medium (2 sectors face economic impacts; score of 2) Low (1 sector impacted; score of 1) No sector faces economic impact (score of 0)
5	Pandemic or epidemic potential	Has the disease caused an epidemic or pandemic in the past?	Disease has previously caused a pandemic worldwide (score of 2) Disease has previously caused an epidemic worldwide (score of 1) Neither is true about the disease (score of 0)

^a^CFR: case fatality rate.

### Step 3: National Multistakeholder Zoonotic Disease Prioritization Workshop

A 3-day national multistakeholder zoonotic disease prioritization workshop was held in Jaipur, Rajasthan, from February 10 to 12, 2020. This workshop included the 50 facilitators, advisors, and voting members described previously, all experts in the fields of epidemiology, microbiology, parasitology, public health, veterinary public health, environmental health, and international health (Textbox 1). The day 1 agenda included the finalization of the weightage and ranking of criteria, the day 2 agenda included the finalization of zoonotic disease rankings, and the day 3 agenda included a workshop summary and action plan (Figure 1).

### Step 4: Ranking of the Criteria and Weighted Score Calculation Through AHP

On the first day of the workshop, the participants familiarized themselves with AHP, which was used to rank criteria and develop a weighted score for each criterion using the OHZDP tool [12].

All the workshop participants were initially divided into 6 sector-specific groups of 6 to 7 participants, a modification of the CDC OHZDP methods. These groups ranked the 5 criteria finalized in step 2 to generate 6 sets of criteria rankings. The OHZDP tool was then used to compute the average ranking from the 6 groups’ inputs and generate the final rankings and criteria weights.

### Step 5: Scoring and Final Ranking of Weighted Criteria for Zoonotic Disease Prioritization

The voters, advisors, and facilitators were then reorganized into 6 heterogenous groups of representative stakeholders. Each group included a moderator from the US CDC (India) or the American Society for Microbiology. Each of these groups was provided with a list of 40 zoonotic diseases and a questionnaire to facilitate the scoring of each categorical question. They were also provided with references for each criterion identified from the literature review. Question scores were then entered into the OHZDP tool to calculate final scores for each zoonotic disease, normalize these scores, and generate the final ranking for each of the 40 listed diseases. The OHZDP process incorporated decision tree analysis to calculate the final weighted score by multiplying the weights of each criterion with the score assigned to each question. A consistency ratio of 0.1 or less was considered satisfactory [28]. We used a Microsoft Excel–based program for AHP to rank the criteria and calculate the consistency ratio by conducting pairwise comparisons of the criteria. For diseases for which participants felt that available data were too limited for scoring to be conducted, ranks were given using simple decision rules based on heuristic knowledge of the existing capacity of health care systems, vaccination, and infrastructure in India. Ranks given using simple decision rules were confirmed through expert consensus within the groups. While this qualitative method had certain limitations in terms of its subjectivity, this was partially overcome by averaging the ranks from the 6 groups and using the best available quantitative and qualitative data for expert consensus ranking.

Sensitivity analysis was conducted to test the validity of the prioritization results based on the impact of input variables (weighted criteria and scoring of individual zoonotic diseases). In the first stage of sensitivity analysis, the 5 criteria were given an equal weight of 1 each to obtain normalized scores. In the next stage, scores for each of the zoonotic diseases were obtained by sequentially removing each of the 5 criteria. The Pearson correlation was used to assess the relationship between normalized scores and the adjusted scores used to assess the impact of criteria weightage and their contribution to disease prioritization ranks. The Pearson correlation coefficient was considered significant at *P*<.05. The analysis was conducted in the Stata software (version 15.1; StataCorp).

### Step 6: Discussion of the Way Forward

The workshop concluded with a discussion on a road map for the prevention and control of identified priority zoonotic diseases, as well as overall areas for improvement, including the development of a national One Health action plan and robust linkages across all sectors.

### Ethical Considerations

This workshop did not require ethics approval or written informed consent because it did not involve research on human participants as defined by institutional and national regulations, and only anonymized aggregate data are reported. Participating experts did not receive any financial or other material compensation for attending the workshop.

## Results

The 40 zoonotic diseases (Textbox 2) were ranked using 5 criteria (Table 1) by 6 groups. The ranking of the criteria conducted by the individual groups using AHP and their consistency ratios are presented in Table 2.

**Table 2:** Group ranking of criteria for prioritizing zoonotic diseases using the analytic hierarchy process.

Criterion	Group 1 score^a^	Group 2 score^a^	Group 3 score^a^	Group 4 score^a^	Group 5 score^a^	Group 6 score^a^	Overall weightage
Severity of the disease in humans (0-3)	0.18	0.35	0.46	0.28	0.35	0.05	0.45
Capacity for prevention and control in humans and the animal health sector (0-3)	0.56	0.35	0.06	0.20	0.39	0.49	0.22
Potential of transmission (0-3)	0.15	0.09	0.12	0.28	0.05	0.16	0.19
Economic burden of the disease (0-3)	0.05	0.10	0.24	0.07	0.15	0.08	0.18
Pandemic or epidemic potential (0-2)	0.06	0.09	0.12	0.18	0.05	0.22	0.18
Consistency ratio^b^	0.06	0.05	0.05	0.08	0.09	0.09	N/A^c^

^a^Score obtained during the analytic hierarchy process (individual group ranking).

^b^A consistency ratio of <0.1 was acceptable.

^c^N/A: not applicable.

The following selected criteria, in the order of the value of their respective weights, were used to rank zoonotic diseases:

1. Severity of the illness in humans (0.45)
2. Capacity for prevention and control in humans and the animal health sector (0.22)
3. Potential for introduction or increased transmission in India (0.195)
4. Economic burden of the disease (0.188)
5. Pandemic potential (0.187)

The distribution of calculated weight and rank for each of the 5 criteria from each of the 6 groups are provided in Table 3. By multiplying these weights with the disease scores given by each heterogenous group, a final normalized score was obtained for each disease (Table 4).

**Table 3:** Definition and final weightage of the criteria identified for zoonotic disease prioritization during the national One Health workshop (February 2020).

Criterion	Questions asked	Response options and score
Severity of the disease in humans (weight=0.45)	Is the disease present in India? What is the CFR^a^ in humans?	Disease present, high CFR (≥5%; score of 3) Disease present, low CFR (<5%; score of 2) Disease not known to be present, high CFR (≥5%; score of 1) Disease not known to be present, low CFR (<5%; score of 0)
Capacity for prevention and control (weight=0.22)	Do measures exist for prevention and for control?	Both prevention and control capacity (score of 3) Prevention but no capacity for control (score of 2) No prevention but capacity for control exists (score of 1) Neither prevention nor control capacity (score of 0)
Potential for introduction or increased transmission in India (weight=0.195)	(1) Does the disease have a feasible transmission pathway to India? (2) Has it been detected in India? (3) Has there been detection and spread in 5 or more new countries, regions, or states?	The response to all 3 questions is yes (score of 3) The response to 2 of the 3 questions is yes (score of 2) The response to 1 of the 3 questions is yes (score of 1) The response to none of the questions is yes (score of 0)
Economic burden of the disease (weight=0.188)	Does the disease cause economic impacts (variables include trade restrictions, decreased animal production, impact on outdoor recreation or tourism, intervention costs, or other secondary impacts [ecological impact])?	High (3 or more sectors face economic impacts; score of 3) Medium (2 sectors face economic impacts; score of 2) Low (1 sector impacted; score of 1) No sector faces economic impact (score of 0)
Pandemic or epidemic potential (weight=0.187)	Has the disease caused an epidemic or pandemic in the past?	Disease has previously caused a pandemic worldwide (score of 2) Disease has previously caused an epidemic worldwide (score of 1) Neither is true about the disease (score of 0)

^a^CFR: case fatality rate.

**Table 4:** Normalized scores for the 40 zoonotic diseases ranked based on the One Health Zoonotic Disease Prioritization process.

Disease	Normalized score
Zoonotic influenza (zoonotic influenza A viruses)	1
Anthrax	0.9318421
Japanese encephalitis	0.9146899
Leptospirosis	0.8946276
Brucellosis	0.8908413
Dengue fever	0.873689
Rabies	0.8636843
Scrub typhus	0.8460808
Plague	0.8447246
CCHF^a^	0.8114685
Zoonotic tuberculosis	0.793719
Nipah virus infection	0.793719
KFD^b^	0.7850176
Listeriosis	0.7765668
Salmonellosis	0.7704677
Echinococcosis	0.7677194
Campylobacteriosis	0.7533154
Q fever	0.7355659
Leishmaniasis	0.7191725
Glanders	0.7084089
West Nile fever	0.7079577
Lyme disease	0.6991103
Yellow fever	0.689026
Shiga toxin–producing *Escherichia coli*	0.6847063
Cysticercosis	0.6505634
Schistosomiasis	0.6505634
Toxoplasmosis	0.6505634
Trypanosomiasis	0.6453343
Melioidosis	0.6397998
Psittacosis	0.6397998
Cryptococcosis	0.6397998
Histoplasmosis	0.6397998
Blastomycosis	0.6397998
Bartonellosis (cat-scratch disease)	0.6305012
MERS-CoV^c^	0.5285279
SARS^d^	0.5285279
Ebola virus disease	0.5285279
Novel coronavirus	0.5285279
Tularemia	0.484171
Lassa fever	0.3865318

^a^CCHF: Crimean-Congo hemorrhagic fever.

^b^KFD: Kyasanur forest disease.

^c^MERS-CoV: Middle East respiratory syndrome–related coronavirus.

^d^SARS: severe acute respiratory syndrome.

**Figure 2:**
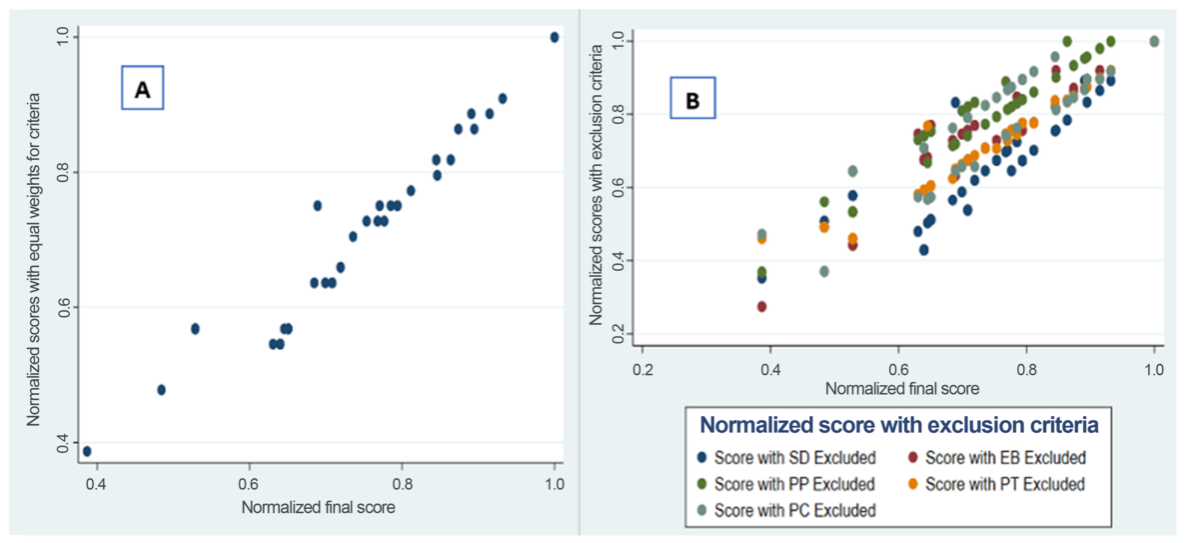
A comparison of (A) normalized scores obtained from the weighted criteria and (B) equal weights excluding each of the 5 criteria. EB: economic burden of disease; PC: capacity for prevention and control; PP: pandemic or epidemic potential; PT: potential of transmission; SD: severity of disease in humans.

As shown in Figure 2, normalized scores were highly consistent across weighting methods. Panel A indicates a strong correlation between weighted and unweighted criteria (*r*=0.95). Panel B shows similarly strong correlations (*r*=0.84-0.96) when each criterion was excluded individually. The sensitivity analysis revealed only limited changes in disease rankings, with the top ten diseases remaining largely robust across scenarios.

After the completion of the prioritization process, the NCDC facilitated a discussion on a future road map for One Health in India. There was broad consensus on establishing clear communication channels between sectors and stakeholders; developing a joint reporting format; establishing a linked laboratory network across India at the state, district, and subdistrict levels; conducting joint surveillance and risk assessment; and institutionalization and implementation of practical One Health coordination mechanisms through the identification of clear roles and responsibilities for different ministries and levels of government.

## Discussion

### Principal Findings

The methodology followed a modified OHZDP process to accommodate India’s context, ensuring inclusivity of stakeholders and harmonization with the existing surveillance and health system structures. The first modification was the selection of criteria before the workshop. The criteria, their responses, and associated scores were finalized 2 days before the workshop through a videoconference led by the NCDC. This ensured smooth workshop progression but limited input from all stakeholders. However, presenting the criteria during the workshop mitigated this limitation, and the ranking process proceeded without further suggestions. The second modification involved sector-specific grouping for criteria ranking. To manage the large number of voting members, they were divided into 6 sector-specific groups. Each group discussed and ranked the criteria, and the results were aggregated for AHP analysis. This modification promoted informed discussions, minimized score variation, and ensured a consistency ratio below the acceptable threshold of 0.1, confirming the process’s integrity. The use of AHP to assign weights and rank criteria provided a transparent, semiquantitative framework that allowed participants to incorporate both quantitative data and expert judgment. Sensitivity analysis validated the robustness of the prioritization as minimal variations were observed in the ranking of the top 10 diseases despite changes in criteria weights. This finding underscores the consistency and reliability of the process and affirms that the prioritization outcomes were not unduly influenced by any single criterion.

Two criteria—severity of the illness in humans and capacity for prevention and control in humans and the animal health sector—received the highest weights, reflecting participants’ emphasis on public health impact and feasibility of intervention. Diseases such as zoonotic influenza, anthrax, and Japanese encephalitis were ranked highly, consistent with their historical and epidemiological relevance to India. These results align with findings from similar OHZDP exercises in other countries, including Bhutan and Uganda, where disease severity and prevention capacity were also key determinants [29,30]. Previous efforts at the local level [25] identified rabies, brucellosis, avian influenza (H5N1), influenza A (H1N1), and CCHF as the highest priority. That list closely resembles the results of this national-level workshop with the exception of avian influenza, which was not included in this workshop.

This prioritization exercise also revealed the utility of combining evidence-based scoring with expert consensus to address data limitations that often constrain disease prioritization in low- and middle-income countries.

The outcomes of this study have direct programmatic implications. The prioritized list serves as a strategic foundation for strengthening surveillance, laboratory capacity, and intersectoral coordination for zoonotic diseases in India. Furthermore, these findings can inform the development of state-level prioritization exercises and One Health action plans, thereby supporting the operationalization of the National One Health Programme for Prevention and Control of Zoonoses.

Limitations of the prioritization process include potential underreporting of certain zoonotic diseases and gaps in data availability, which may have influenced expert scoring. However, these were mitigated through an extensive literature review and expert consensus. Despite these limitations, the structured use of AHP, high consistency ratios, and minimal rank variation in sensitivity analysis together demonstrate the robustness of the findings.

In summary, this study not only identifies the zoonotic diseases of highest national concern but also demonstrates the applicability and adaptability of the OHZDP process in a complex and diverse health system such as India’s. The results provide an evidence-based foundation for future multisectoral action, capacity building, and surveillance system strengthening in alignment with India’s One Health vision. Through the conduct of this prioritization workshop, India has demonstrated its commitment to One Health through interministerial collaboration and a multidisciplinary, multisectoral approach to prioritization, demonstrating “functionality with bridges and fluidity” [31] in action.

### Conclusions

Zoonotic influenza (zoonotic influenza A viruses) was the highest-ranked zoonosis in this workshop. Overcoming the programmatic silos that currently exist within different government agencies and institutions will be a major challenge in operationalizing One Health in India; however, this workshop demonstrated that such coordination is possible. This prioritization conducted at the national level has the potential to catalyze such efforts at the state and local levels across India by fostering the communication, collaboration, cooperation, and coordination necessary to make meaningful progress. Given the diversity in geography and prevalence of communicable diseases in different parts of India, states may be interested in taking up similar exercises to further streamline their needs and resource use. State action plans developed under the guidance of the National One Health Programme for Prevention and Control of Zoonoses will help states achieve the One Health goals outlined by the National One Health Programme for Prevention and Control of Zoonoses.

## Abbreviations

AHP: analytic hierarchy process
CCHF: Crimean-Congo hemorrhagic fever
CDC: Centers for Disease Control and Prevention
IDSP: Integrated Disease Surveillance Programme
NCDC: National Centre for Disease Control
OHZDP: One Health Zoonotic Disease Prioritization

## References

[1] Chitale VS, Behera MD, Roy PS (2014). Future of endemic flora of biodiversity hotspots in India. PLoS One.

[2] Morens DM, Folkers GK, Fauci AS (2004). The challenge of emerging and re-emerging infectious diseases. Nature.

[3] Neiderud CJ (2015). How urbanization affects the epidemiology of emerging infectious diseases. Infect Ecol Epidemiol.

[4] Rohr JR, Barrett CB, Civitello DJ, Craft ME, Delius B, DeLeo GA, Hudson PJ, Jouanard N, Nguyen KH, Ostfeld RS, Remais JV, Riveau G, Sokolow SH, Tilman D (2019). Emerging human infectious diseases and the links to global food production. Nat Sustain.

[5] Jones KE, Patel NG, Levy MA, Storeygard A, Balk D, Gittleman JL, Daszak P (2008). Global trends in emerging infectious diseases. Nature.

[6] Tripartite and UNEP support OHHLEP's definition of "One Health". World Health Organization (WHO).

[7] Jones B, McKeever DJ, Grace D, Pfeiffer DU, Mutua F, Njuki J, McDermott J (2011). Zoonoses (Project 1): wildlife/domestic livestock interactions. UK Department for International Development and ILRI.

[8] Gates B (2020). Responding to COVID-19 - a once-in-a-century pandemic?. N Engl J Med.

[9] No authors listed (2020). Emerging zoonoses: a one health challenge. EClinicalMedicine.

[10] Cunningham AA, Daszak P, Wood JL (2017). One Health, emerging infectious diseases and wildlife: two decades of progress?. Philos Trans R Soc Lond B Biol Sci.

[11] One Health. Center for Disease Control and Prevention (CDC).

[12] Rist CL, Arriola CS, Rubin C (2014). Prioritizing zoonoses: a proposed one health tool for collaborative decision-making. PLoS One.

[13] Stebler N, Schuepbach-Regula G, Braam P, Falzon L (2015). Use of a modified Delphi panel to identify and weight criteria for prioritization of zoonotic diseases in Switzerland. Prev Vet Med.

[14] Kadohira M, Hill G, Yoshizaki R, Ota S, Yoshikawa Y (2015). Stakeholder prioritization of zoonoses in Japan with analytic hierarchy process method. Epidemiol Infect.

[15] Munyua P, Bitek A, Osoro E, Pieracci EG, Muema J, Mwatondo A, Kungu M, Nanyingi M, Gharpure R, Njenga K, Thumbi SM (2016). Prioritization of Zoonotic diseases in Kenya, 2015. PLoS One.

[16] Sekamatte M, Krishnasamy V, Bulage L, Kihembo C, Nantima N, Monje F, Ndumu D, Sentumbwe J, Mbolanyi B, Aruho R, Kaboyo W, Mutonga D, Basler C, Paige S, Barton Behravesh C (2018). Multisectoral prioritization of zoonotic diseases in Uganda, 2017: a One Health perspective. PLoS One.

[17] Kheirallah KA, Al-Mistarehi AH, Alsawalha L, Hijazeen Z, Mahrous H, Sheikali S, Al-Ramini S, Maayeh M, Dodeen R, Farajeh M, Masadeh N, Alemam A, Alsulaiman J, Samhouri D (2021). Prioritizing zoonotic diseases utilizing the One Health approach: Jordan's experience. One Health.

[18] Completed OHZDP workshops. Center for Disease Control and Prevention (CDC).

[19] Ihekweazu C, Michael CA, Nguku PM, Waziri NE, Habib AG, Muturi M, Olufemi A, Dzikwi-Emennaa AA, Balogun MS, Visa TI, Dalhat MM, Atama NC, Umeokonkwo CD, Mshelbwala GM, Vakuru CT, Kabir J, Okolocha EC, Umoh JU, Olugasa B, Babalobi O, Lombin L, Cadmus S, Nigeria Zoonotic Diseases Prioritization Group (2021). Prioritization of zoonotic diseases of public health significance in Nigeria using the one-health approach. One Health.

[20] One Health Zoonotic disease prioritization (OHZDP). Center for Disease Control and Prevention (CDC).

[21] Weekly outbreaks. Integrated Disease Surveillance Programme (IDSP).

[22] Bhatt PN, Rodrigues FM (1967). Chandipura: a new Arbovirus isolated in India from patients with febrile illness. Indian J Med Res.

[23] Sudeep AB, Gurav YK, Bondre VP (2016). Changing clinical scenario in Chandipura virus infection. Indian J Med Res.

[24] Cecilia D (2014). Current status of dengue and chikungunya in India. WHO South East Asia J Public Health.

[25] Yasobant S, Saxena D, Bruchhausen W, Memon FZ, Falkenberg T (2019). Multi-sectoral prioritization of zoonotic diseases: One Health perspective from Ahmedabad, India. PLoS One.

[26] O'Cathain A, Murphy E, Nicholl J (2008). The quality of mixed methods studies in health services research. J Health Serv Res Policy.

[27] Ben-Assuli O, Kumar N, Arazy O, Shabtai I (2020). The use of analytic hierarchy process for measuring the complexity of medical diagnosis. Health Informatics J.

[28] Pant S, Kumar A, Ram M, Klochkov Y, Sharma H (2022). Consistency indices in analytic hierarchy process: a review. Mathematics.

[29] Tripartite supported national workshops on one health zoonotic diseases prioritization and joint risk assessment under pandemic fund in Bhutan. World Health Organization (WHO).

[30] (2017). One Health Zoonotic disease prioritization for multi-sectoral engagement in Uganda: workshop summary. Center for Disease Control and Prevention (CDC).

[31] Panda S, Bhargava B, Gupte M (2021). One world One Health: widening horizons. Indian J Med Res.

